# Solitary splenic tuberculosis: a case report and review of the literature

**DOI:** 10.1186/s12957-016-0905-6

**Published:** 2016-06-01

**Authors:** Sai-Feng Lin, Lei Zheng, Lei Zhou

**Affiliations:** Department of Ultrasound, the First Affiliated Hospital of Wenzhou Medical University, Wenzhou, 325000 Zhejiang Province China; Department of Gastrointestinal Surgery, the Second Affiliated Hospital of Wenzhou Medical University, Wenzhou, 325000 Zhejiang Province China

**Keywords:** Splenic tuberculosis

## Abstract

**Background:**

Tuberculosis remains one of the most prevalent and fatal infectious diseases in spite of considerable improvements in medical science. Tuberculosis is an important health problem in developing countries. There are few cases of solitary splenic tuberculosis reported in the literature internationally. Solitary splenic tuberculosis is extremely rare and is mostly seen in individuals with immunosuppression. Patients susceptible to acquiring splenic tuberculosis usually have some risk factors such as immunosuppression, pyogenic infections, splenic abnormalities, spleen trauma, sickle cell disease, and so on (Basa JV, Singh L, Jaoude WA, Sugiyama G, Int J Surg 8C:117–119,2015).

**Case presentation:**

Here we report a case of surgically confirmed mass-forming solitary splenic tuberculosis in a 64-year-old woman who presented with abdominal discomfort for two months, but with no other symptoms. Laboratory data provided no specific information for diagnosis. Abdominal ultrasonography revealed a large hypoechoic lesion within the spleen. Computed tomography scan of the abdomen showed a solitary hypodense lesion. A diagnosis of solitary splenic tuberculosis was confirmed after a splenectomy was performed and histopathological examination revealed splenic tuberculosis.

**Conclusions:**

Solitary splenic tuberculosis is rare and associated with an immunocompetent patient is extremely rare. It is hard to correctly diagnose it by US or CT scan.

## Background

Despite medical improvement in the diagnosis and treatment of infectious diseases over the years, tuberculosis continues to be a major health problem in developing countries. There are few cases of solitary splenic tuberculosis reported in the literature internationally, especially single large mass-forming splenic tuberculosis. Splenic tuberculosis is extremely rare. Approximately 15–20 % of all cases of tuberculosis are extrapulmonary; of these 3–11 % are abdominal. Splenic tuberculosis is very unusual with only a few case reports. In a series evaluated retrospectively, the rate of splenic tuberculosis was reported as 8 %, while in the literature the rate of micronodular type of involvement was reported at around 5 %. Splenic tuberculosis presents with diverse clinical symptoms. The most common symptoms that patients present with are fever (82.3 %), fatigue and weight loss (44.12 %), and splenomegaly (13.2–100 %). Therefore, it is likely to be misdiagnosed as carcinoma of the spleen, metastases tumor, lymphoma, hemangioma, or splenic abscess. The misdiagnosis rate is high if there is no tuberculosis history in any other organs. Through the literature retrieval, at present, there are no reports about splenic tuberculosis mortality rate data.

In this case report, we present the case of a 64-year-old woman solitary splenic tuberculosis and with no other significant past medical history.

## Case presentation

A 64-year-old Chinese woman presented to our outpatient service with abdominal discomfort associated with nausea for two months, but no other symptoms. There was no history of fever, cough, chest or abdominal pain, night sweats, weight loss, or anorexia. Her history included a 4-year cholecystectomy. Her medical history did not include any tuberculosis and human immunodeficiency virus (HIV) infection. On physical examination, her body temperature was 36.5 °C. Palpation of the abdomen revealed epigastric tenderness and no hepatosplenomegaly. Routine blood analysis revealed mild anemia, hemoglobin level was 100 g/L, blood platelet count was 242 × 109/L. Liver function tests were within normal limits (total bilirubin: 5 mg/dL, direct bilirubin: 2 mg/dL, indirect bilirubin: 3 mg/dL, total protein: 68.1 gm/dL, albumin: 34.7gm/dL, SGOT: 17 U/L, SGPT: 12 U/L). Tumor markers were within normal limits and HIV antibody reaction was negative. Chest radiography and cardiac ultrasound (US) revealed no abnormalities.

**Fig. 1:**
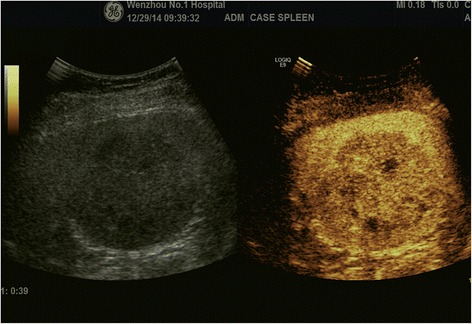
Ultrasound showing splenic hypoechoic lesion appearing with a rounded, clear boundary, heterogeneous enhancement, and centrally irregular necrosis

Abdominal US showed the spleen with a large hypoechoic lesion. The mass size was 6.9 × 6.9 × 6.7 cm with regular margins and homogenous internal echo. Contrast-enhanced US showed that splenic hypoechoic lesion appeared with a rounded, clear boundary, heterogeneous enhancement, and centrally irregular necrosis. Our diagnosis was lymphoma (Fig. 1).

**Fig. 2:**
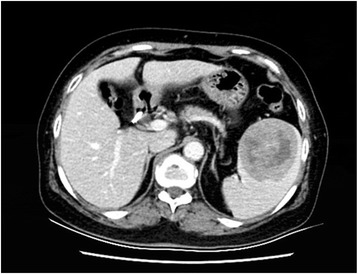
Abdominal computed tomography scan revealed a big, single hypodense, 6.4 × 6.5 cm in size lesion in the spleen. Clear boundary, heterogeneous enhancement, and centrally irregular necrosis

Abdominal computed tomography (CT) scan showed a large hypodense lesion (6.4 × 6.5 cm) on the spleen with an unclear boundary which became clear after enhanced scanning and heterogeneous lesion enhancement. Centrally irregular necrosis can be seen in the mass. Our diagnosis was inflammatory pseudotumor (Fig. 2).

The patient did not have a magnetic resonance imaging examination before surgery.

**Fig. 3:**
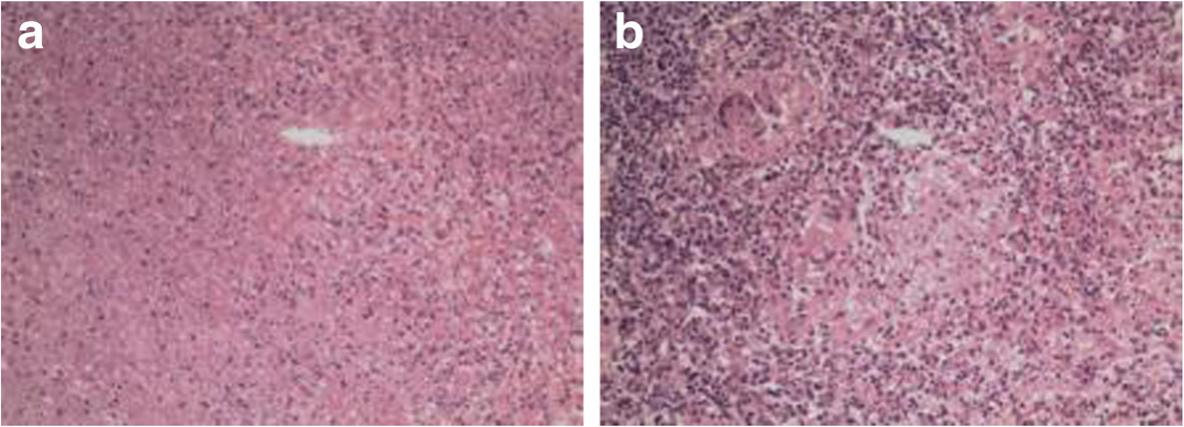
**a**, **b** Splenic granulomatous inflammation with large inflammatory necrosis and caseation necrosis. (Hematoxylin and eosin stained technique, middle-multiplications)

Due to the position of the mass and the risk of gastrointestinal tract injury and tumor bleeding, needle biopsy of the spleen mass was excluded. The patient therefore underwent laparoscopic splenectomy for diagnostic and therapeutic purposes. The histopathological report showed splenic granulomatous inflammation with large inflammatory necrosis and caseation necrosis. We finally diagnosed splenic tuberculosis. After surgery, the patient was started on quadruple anti-tuberculosis therapy (rimifon, streptomycin, rifapin, pyrazinamide, and ethambutol) (Fig. 3a and b).

Three months after splenectomy, the patient presented to our outpatient service for re-examination. Routine blood analysis revealed mild anemia, hemoglobin level was 108 g/L, blood platelet count was 258 × 10^9^/L. Liver function tests were within normal limits, purified protein derivative test was negative. Chest radiography and abdominal CT scan revealed no abnormalities.

## Discussion

Tuberculosis is a multi-system disease and pulmonary tuberculosis is the most common manifestation. Extra pulmonary disease accounts for almost 15–20 % of all tuberculosis [1]. Splenic tuberculosis was first described in the literature in 1846 by Coley. This unusual form of splenic tuberculosis is the primary involvement which is rarely reported in the literature. Patients infected with HIV or who are immunocompromised have been revealed to be a high risk for splenic tuberculosis. Many reported cases of splenic tubercular abscess are found to have underlying HIV infections [2]. Splenic involvement therefore had been thought to be seen only in immunocompromised individuals. However, there are few case reports of splenic tuberculosis in immunocompetent patients [3]. Sharma et al. and Gupta et al., respectively, reported rare cases of splenic abscess in an immunocompromised and an immunocompetent patient [4, 5]. As we present here, this case is exceptionally rare. The patient neither had a history of tuberculosis nor showed any indication of tuberculosis in the other organs. No immunosuppressive condition that could cause such infection was demonstrated. Clinical and routine laboratory findings were non-specific. The most common symptoms that patients present with are fever (82.3 %), fatigue and weight loss (44.12 %), and splenomegaly (13.2–100 %) [6]. There are no specific symptoms for establishing the diagnosis of splenic tuberculosis. In our case, the chief complaint symptom was abdominal discomfort, without fever or weight loss. Splenic tuberculosis in an immunocompetent individual is very rare and poses a difficult diagnosis.

Despite the reliability of common methods such as US and CT in distinguishing such lesions, from primary or metastatic tumor of the spleen, it has its limitations. The misdiagnosis rate is high if there is no tuberculosis history in other organs.

In our case, a single and solid mass was seen on the spleen with contrast-enhanced US. The splenic hypoechoic lesion appeared with a rounded, well-demarcated, heterogeneous enhancement, and centrally irregular necrosis. At first, we misdiagnosed this as lymphoma.

Contrast-enhanced CT showed a splenic hypodense lesion appearing with a rounded, clear boundary, heterogeneous enhancement, and irregular necrosis, which at first we misdiagnosed as an inflammatory pseudotumor. There are many situations that may have presentations of single and solid masses, splenic hypodense lesions on CT, and hypoechoic lesions on US such as malignant lymphoma, metastatic tumor, acute leukemia, hemangioma, or even infectious diseases. Our patient was misdiagnosed at first.

In almost all the reported cases, the diagnosis was first made by radiological findings followed by pathological examination of a fine-needle aspirate, splenic biopsy, or splenectomy. Needle biopsy of the spleen is an important method of diagnosis. If biopsy fails, laparoscopic splenectomy has been used in the diagnosis of splenic tuberculosis and has proved to be a minimally invasive approach. Surgery remains the gold standard for definitive diagnosis of such cases with an undefined etiology.

In our case, because of the position of the mass and the risk of gastrointestinal tract perforation and tumor bleeding by needle biopsy of the spleen mass, laparoscopic splenectomy was carried out.

So far, histopathological examination is still an ideal method to confirm the diagnosis. There are five types of pathomorphological classifications for splenic tuberculosis including miliary tuberculosis, nodular tuberculosis, tuberculous spleen abscess, calcific tuberculosis, and mixed type tuberculosis.

Like the treatment of pulmonary tuberculosis, antitubercular treatment is the primary modality in treating splenic tuberculosis. Splenic tuberculosis must be carried out in accordance with the following principles: timely treatment in combination, regularly and properly through the whole course whether or not an operation is performed [7].

## Conclusions

Solitary splenic tuberculosis is rare and associated with an immunocompetent patient is extremely rare. It is hard to correctly diagnose it by US or CT scan. We hope that more detailed information about the differential diagnosis, especially non-invasive imaging techniques, will be constantly updated.

## Abbreviations

CT, computed tomography; HIV, human immunodeficiency virus; US, ultrasound

## References

[1] Hamizah R, Rohana AG, Anwar SA, Ong TZ, Hamazaini AH, Zuikarnaen AN (2007). Splenic tuberculosis presenting as pyrexia of unknown origin. Med J Malaysia.

[2] Pramesh CS, Tamhankar AP, Rege SA, Shah SR (2002). Splenic tuberculosis and HIV-1 infection. Lancet.

[3] Chandra S, Srivastava DN, Gandhi D (1999). Splenic tuberculosis: an unusual sonographic presentation. Int J Clin Pract.

[4] Sharma S, Dey AB, Agarwal N, Nagarkar KM, Gujral S (1999). Tuberculosis: a rare cause of splenic abscess. J Assoc Physicians India.

[5] Gupta A, Hunjan PS, Jain SK, Kaza RC, Kumar V (2006). Tubercular splenic abscess in an immunocompetent patient: a rare entity. Southeast Asian J Trop Med Public Health.

[6] Rhazal F, Lahlou MK, Benamer S, Daghri JM, Essadel E, Mohammadine E (2004). Splenomegaly and splenic pseudotumor due to tuberculosis: six new cases. Ann Chir.

[7] Zhan F, Wang C-J, Lin J-Z, Zhong P-J, Qiu W-Z, Lin H-H (2010). Isolated splenic tuberculosis: A case report. World J Gastrointest Pathophysiol.

